# Alcohol intake and risk of thyroid cancer in the NIH-AARP Diet and Health Study

**DOI:** 10.1038/sj.bjc.6605337

**Published:** 2009-09-29

**Authors:** C L Meinhold, Y Park, R Z Stolzenberg-Solomon, A R Hollenbeck, A Schatzkin, A Berrington de Gonzalez

**Affiliations:** 1 Division of Cancer Epidemiology and Genetics, National Cancer Institute, National Institutes of Health, Rockville, MD, USA; 2 AARP, Washington, DC, USA

**Keywords:** thyroid cancer, alcohol drinking, cohort study, epidemiology

## Abstract

**Background:**

Certain studies suggest that alcohol may reduce the risk of thyroid cancer in women, but the effect in men remains unclear.

**Methods:**

We analysed the association between alcohol and thyroid cancer in a large (*n*=490 159) prospective NIH-AARP Diet and Health Study with self-reported beer, wine, and liquor intakes.

**Results:**

Over 7.5 years of follow-up (median), 170 men and 200 women developed thyroid cancer. Overall, the thyroid cancer risk decreased with greater alcohol consumption (⩾2 drinks per day *vs* none, relative risk=0.57, 95% CI 0.36–0.89, *P*-trend=0.01).

**Conclusions:**

These results suggest a potential protective role for alcohol consumption in thyroid cancer.

The rapid increase in thyroid cancer incidence in the United States over the past three decades may be related to changes in certain environmental exposures, as well as increased surveillance and more widespread use of sensitive diagnostic tools (Enewold *et al*, 2009). However, despite many retrospective studies, few risk factors have been established other than childhood exposure to ionizing radiation (Dal Maso *et al*, 2009).

Certain studies have suggested an inverse association between alcohol and thyroid cancer risk, but this may be confounded by cigarette smoking, which is correlated with alcohol consumption and has been inversely related with thyroid cancer (Mack *et al*, 2003; Jee *et al*, 2004; Nagano *et al*, 2007). However, no association was observed in a pooled analysis of 14 case–control studies after adjusting for smoking (Mack *et al*, 2003), or in two prospective studies with fewer than 200 cases (Iribarren *et al*, 2001; Navarro Silvera *et al*, 2005), although these studies were consistent with a small reduction in risk. A larger prospective study in women (421 cases) showed a clear reduction in risk with greater alcohol consumption (Allen *et al*, 2009). Although it remains unclear whether an association between alcohol and thyroid cancer exists independently of smoking, there is also a need to investigate effect modification by sex, given the three-fold higher incidence of thyroid cancer in women compared with men (Jemal *et al*, 2008).

Given the evidence of a possible association and a recent decrease in alcohol consumption in the United States (Zhang *et al*, 2008; Kerr *et al*, 2009), we analysed the association between alcohol intake and the risk of thyroid cancer and its sub-types in a large US prospective study of over 490 000 participants, including more than 292 000 men.

## Materials and methods

The NIH-AARP Diet and Health Study began in 1995–1996 when a questionnaire was mailed to AARP members, aged 50–71 years, residing in six US states (California, Florida, Louisiana, New Jersey, North Carolina, and Pennsylvania) and two metropolitan areas (Atlanta and Detroit; Schatzkin *et al*, 2001). Participant mailings and annual linkage to 11 state cancer registries and the National Death Index provide information on cancer and mortality outcomes. The NIH-AARP Study was approved by the special studies institutional review board of the US National Cancer Institute. Further details on this cohort have been previously described (Schatzkin *et al*, 2001).

Among 567 169 questionnaire respondents, we excluded individuals who provided duplicate questionnaires (*n*=179), withdrew from the study (*n*=6), moved outside the study area or died before follow-up (*n*=617), were proxy respondents (*n*=15 760), had prevalent cancer except non-melanoma skin cancer (*n*=51 193) or end-stage renal disease at baseline (*n*=997), had cancer identified only from death records (*n*=3876), or had missing or extreme (greater than twice the interquartile range of sex-specific transformed intake above the 75th or below the 25th percentile) caloric intake (*n*=4 382). The analytic cohort included 490 159 participants (292 101 men and 198 058 women).

We defined incident thyroid cancer cases as first invasive, malignant neoplasm diagnosed during follow-up, and classified histology according to International Classification of Diseases for Oncology, Third Edition codes (C739) (Fritz *et al*, 2000).

The self-administered baseline questionnaire inquired about health-related behaviours, demographics, and anthropometric characteristics. A supplementary food frequency questionnaire elicited information on regular dietary habits over the past year, including serving sizes and frequency of intake of beer during the summer and rest of the year, wine, and liquor. On the basis of this information, alcohol consumption was standardised according to the MyPyramid Servings database of the US Department of Agriculture. One serving (drink) corresponds to 12 fluid ounces of beer (12.96 g ethanol), 5 fluid ounces of wine (13.72 g ethanol), and 1.5 fluid ounces of 80-proof distilled spirits (13.93 g ethanol).

Cox proportional hazards models with attained age as the time metric were used to estimate relative risks (RR) and 95% confidence intervals (CI) for thyroid cancer in all participants and by sex. Models were additionally adjusted for sex, race, smoking status, body mass index (BMI), and family history of cancer. We mutually adjusted for beer, wine, and liquor in models of alcohol type. Medians of each exposure category were modeled as continuous in trend tests. Sub-group analyses were conducted using categories of sex, smoking status, BMI, and thyroid cancer histology. The heterogeneity within sub-groups was assessed using the *Q* statistic (Cochran, 1954). We found no evidence that the proportional hazards assumption was violated.

## Results

The median alcohol intake among participants reporting any alcohol consumption over the past year was 4.5 g per day, or approximately one-third of the amount of a standard alcoholic drink. Over 11% of the cohort (9574 women and 45 149 men) reported consuming two or more alcoholic drinks daily. There were higher proportions of beer or liquor drinkers among the heavier drinking groups, and of wine drinkers among the more moderate drinking group. Compared with non-drinkers, heavier drinkers were more likely to be male, white non-Hispanic, more educated, and to have ever smoked, and less likely to be obese (Table 1).

Over a median 7.5 years of follow-up, 200 women and 170 men were diagnosed with thyroid cancer. Compared with non-drinking, consuming two or more drinks per day was associated with a significantly decreased risk (RR=0.57, 95% CI 0.36–0.89, *P*-trend=0.01; Table 2). Consuming one or more drinks per day of beer was associated with a decreased risk when compared with no beer drinking, particularly in men (RR=0.47, 95% CI 0.22–0.97, *P*-trend=0.03). No clear dose-response associations with wine were observed, but liquor intake was associated with a nonsignificant decreased risk in women.

**Figure 1:**
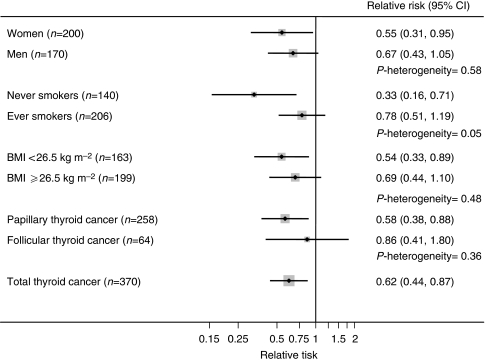
Associations between alcohol intake (⩾1 drink per day *vs* none) and thyroid cancer risk by sex, smoking status, body mass index (BMI), and histological type, using the NIH-AARP Diet and Health Study (*n*=490 159). The models were adjusted for age, sex, race (non-Hispanic white, non-Hispanic black, other, or missing), education (less than high school, high school graduate, some college, college graduate, or missing), smoking status (never, former, current, or missing), body mass index (BMI<25.25−29.9, ⩾30 kg m^−2^ or missing), and family history of cancer (no, yes, or missing).

The strength of the inverse association with alcohol intake was relatively consistent according to sex, smoking status, and BMI categories, as well as histology (Figure 1). It was stronger in participants who never smoked (RR=0.33, 95% CI 0.16–0.71) when compared with ever smokers (RR=0.78, 95% CI 0.51–1.19; *P*-heterogeneity=0.05). Although we observed a clearer inverse association for papillary (RR=0.58, 95% CI 0.38–0.88) as compared with follicular cancer (RR=0.86, 95% CI 0.41–1.80), these results were not significantly different (*P*-heterogeneity=0.36). For every 10 g consumed, the RRs for thyroid cancer were 0.95 (95% CI 0.89–1.01) for men, 0.84 (95% CI 0.71–1.01) for women, and 0.94 (95% CI 0.88–0.99) for men and women combined. The results were not materially altered after excluding the first 2 years of follow-up, when the lightest drinkers were considered as the referent category, or when models were additionally adjusted for smoking intensity (data not shown).

## Discussion

In this large prospective study, we observed that greater alcohol intake was associated with a reduced risk of thyroid cancer in both men and women, independent of cigarette smoking. The inverse association for beer seemed stronger than for wine or liquor, particularly among men in this cohort. In women, we observed a nonsignificant inverse association for liquor intake, but no clear association for other alcohol types. These differences may reflect the smaller range of alcohol intake in women, residual confounding by socioeconomic status or related factors, drinking patterns (i.e., binge drinking), or differences in measurement error by the type of alcohol. Nonetheless, similar dose-response associations with total alcohol intake were observed by sex, and associations by alcohol type were generally in the same inverse direction. Although we observed a clearer reduction in risk for papillary *vs* follicular cancers, this difference was not significant. However, there were only 64 follicular cancers in this study.

Given the strong, positive correlation between alcohol and smoking, and the evidence linking smoking with a reduced risk of thyroid cancer (Mack *et al*, 2003; Jee *et al*, 2004; Nagano *et al*, 2007), residual confounding by smoking may have biased our results away from the null. However, this seems unlikely as a stronger inverse association for alcohol was observed for never when compared with ever cigarette smokers.

In a cross-sectional study using ultrasonography, an inverse association between alcohol consumption and thyroid enlargement and nodularity was observed (Knudsen *et al*, 2001). A positive association between alcohol consumption and thyroid-stimulating hormone (TSH) and an inverse association with serum T3 was also found. Thyroid-stimulating hormone, which is secreted by the pituitary in response to low thyroid hormone levels in the blood, is known to increase the proliferation of follicular thyroid cells in laboratory studies (Williams, 1990), although its effect in humans is not clearly established. Alcohol may protect the thyroid from elevated TSH by inhibiting thyroid hormone metabolism (Knudsen *et al*, 2001). Greater alcohol intake may lead to vitamin deficiencies, impaired folate metabolism, oxidative stress, and DNA damage (Boffetta and Hashibe, 2006); but whether these directly influence the thyroid is unknown.

This is one of the few prospective studies to examine the relation between alcohol and thyroid cancer risk. A reasonably large number of cases allowed for sub-group analyses by sex, BMI, smoking status, and thyroid cancer sub-types; however, there were too few cases to obtain stable RRs in the heavier-drinking categories (⩾2 drinks per day). Participants reported intake during the past year, which obviously does not cover cumulative lifetime drinking, and our results could be attenuated by the inclusion of recent quitters in the reference category of non-drinkers. However, excluding the first 2 years of follow-up did not materially change the results, and a similar inverse dose-response association was observed when the lightest drinkers were the referent group.

In this large prospective study, we found a reduction in thyroid cancer risk in both men and women with a greater consumption of alcohol. The decrease in risk associated with consuming 10 g per day is reasonably consistent between women in this study and in another large prospective study (16 *vs* 25% per 10 g per day; Allen *et al*, 2009). However, the direct public health applications of these findings are limited, considering the evidence linking moderate-to-heavy alcohol consumption with increases in several cancers, among other adverse effects (Boffetta and Hashibe, 2006). Nonetheless, as thyroid cancer incidence is rising, it is important to analyse the influence of diet and lifestyle in its aetiology.

## Figures and Tables

**Table 1:** Study characteristics by alcohol intake (medians or percentage), using the NIH-AARP Diet and Health Study (*n*=490 159)

	**Alcohol (drinks)**				
	**None**	**<1 per week**	**1–6 per week**	**1–2 per day**	**⩾2 per day**
Study participants	119 172	131 726	127 340	57 198	54 723
Alcohol (drinks per day)	0	0.06	0.38	1.34	3.66
Alcohol (g per day)	0	0.79	5.16	17.91	50.84
					
*Drinking pattern (%)*					
Mostly beer drinkers†	N/A	20	27	23	33
Mostly wine drinkers†	N/A	42	36	36	18
Mostly liquor drinkers†	N/A	28	24	36	46
Mixed	N/A	10	13	5	4
Age at entry	63.0	62.4	62.1	63.1	62.6
					
*Sex (%)*					
Women	49	54	34	28	18
Men	51	46	67	72	83
					
*Race/ethnicity (%)*					
White, non-Hispanic	87	91	93	95	95
Black, non-Hispanic	6	4	3	2	2
Other	4	4	3	3	2
Missing	2	1	1	1	1
					
*Education (%)*					
Less than high school	35	28	20	18	20
High school graduate	10	11	9	9	9
Some college	22	24	24	23	24
College graduate	29	35	44	49	45
Missing	4	3	3	2	2
					
*Body mass index (%)*					
<25 kg m^−2^	32	34	36	40	34
25–29.9 kg m^−2^	39	39	44	44	46
⩾30 kg m^−2^	26	24	18	15	18
Missing	3	2	2	2	2
					
*Cigarette smoking status (%)*					
Never	43	41	33	26	18
Former	42	44	53	58	60
Current	11	12	10	12	19
Missing	4	4	4	4	4
					
*Family history of cancer (%)*					
No	46	46	46	46	47
Yes	48	49	49	49	48
Missing	6	5	5	5	5

† Gram intake of specific alcohol type accounts for 750% of total alcohol intake.

**Table 2:** Association between the level of alcohol intake and thyroid cancer risk, using the NIH-AARP Diet and Health Study, (*n*=490 159)

	**Total (*n*=490 159)**			**Women (*n*=198 058)**			**Men (*n*=292 101)**		
	**Cases/person- years**	**RR^a^**	**RR (95% CI)^b^**	**Cases/person- years**	**RR^a^**	**RR (95% CI)^b^**	**Cases/person- years**	**RR^a^**	**RR (95% CI)^b^**
*Alcohol (drinks)*									
None	109/812 376	1.00	1.00 (Reference)	71/405 668	1.00	1.00 (Reference)	38/406 708	1.00	1.00 (Reference)
<1 per week	109/913 627	0.88	0.87 (0.66–1.13)	70/503 876	0.80	0.81 (0.58–1.14)	39/409 751	1.02	0.97 (0.62–1.51)
1–6 per week	96/878 488	0.89	0.87 (0.66–1.16)	43/300 016	0.83	0.84 (0.57–1.24)	53/578 473	0.99	0.90 (0.59–1.38)
1–2 per day	32/393 092	0.67	0.67 (0.44–1.00)	16/176 983	0.52^c^	0.55 (0.31–0.95)^c^	20/282 064	0.76	0.69 (0.40–1.20)
⩾2 per day	24/369 886	0.57	0.57 (0.36–0.89)	—	—	—	20/303 931	0.71	0.64 (0.37–1.12)
*P*-trend			0.01			0.06			0.08
									
*Beer (drinks)* d ^,^ e									
None	195/1 542 426	1.00	1.00 (Reference)	135/896 073	1.00	1.00 (Reference)	60/646 353	1.00	1.00 (Reference)
<1 per week	128/1 111 220	1.02	1.02 (0.79–1.32)	56/398 773	1.01	1.02 (0.72–1.44)	72/712 447	0.99	0.99 (0.66–1.48)
1–6 per week	38/481 530	0.79	0.79 (0.53–1.17)	9/91 697	0.72^f^	0.75 (0.37–1.49)^f^	29/408 543	0.71	0.72 (0.44–1.21)
⩾1 per day	9/232 293	0.40	0.42 (0.21–0.83)	—	—	—	9/213 583	0.43	0.47 (0.22–0.97)
*P*-trend			0.01			0.40			0.03
									
*Wine (drinks)* e ^,^ g									
None	147/1 317 699	1.00	1.00 (Reference)	82/549 427	1.00	1.00 (Reference)	65/768 272	1.00	1.00 (Reference)
<1 per week	145/1 267 510	1.07	1.05 (0.81–1.37)	80/556 231	1.09	1.10 (0.77–1.55)	65/711 279	1.05	0.99 (0.67–1.48)
1–6 per week	61/556 399	1.11	1.09 (0.77–1.54)	30/204 015	1.16	1.15 (0.71–1.86)	31/352 384	1.08	1.00 (0.61–1.63)
⩾1 per day	17/225 863	0.77	0.76 (0.45–1.29)	8/76 870	0.81	0.83 (0.39–1.77)	9/148 993	0.73	0.69 (0.33–1.43)
*P*-trend			0.69			0.62			0.33
									
*Liquor (drinks)* e ^,^ h									
None	187/1 575 603	1.00	1.00 (Reference)	117/725 512	1.00	1.00 (Reference)	70/850 091	1.00	1.00 (Reference)
<1 per week	124/1 145 879	0.93	0.93 (0.71–1.20)	63/493 054	0.78	0.79 (0.56–1.11)	61/652 825	1.20	1.15 (0.77–1.72)
1–6 per week	27/332 326	0.79	0.79 (0.51–1.22)	12/92 127	0.79	0.82 (0.44–1.54)	15/240 199	0.87	0.81(0.44–1.50)
⩾1 per day	32/313 660	1.02	1.02 (0.68–1.53)	8/75 849	0.65	0.70 (0.33–1.47)	24/237 811	1.40	1.25 (0.74–2.11)
*P*-trend			0.80			0.46			0.52

a Adjusted for age and sex.

b Adjusted for age, sex, race (non-Hispanic white, non-Hispanic black, other, or missing), education (less than high school, high school graduate, some college, college graduate, or missing), smoking status (never, former, current, or missing), body mass index (BMI <25, 25–29.9, ⩾30 kgm^−2^, or missing), and family history of cancer (no, yes, or missing).

c Relative risks for ⩾1 drink per day *vs* none.

d One standardized drink of beer is equal to 12.96 g of alcohol.

e Beer, wine, and liquor were mutually adjusted for in age- and sex-adjusted and multivariable-adjusted models.

f Relative risks for ⩾1 drinks per week *vs* none.

g One standardized drink of wine is equal to 13.72 g of alcohol.

h One standardized drink of liquor is equal to 13.93 g of alcohol.
